# ﻿A new species of the *Cymbasoma longispinosum* species-group (Copepoda, Monstrilloida) from the northern South China Sea

**DOI:** 10.3897/zookeys.1264.161966

**Published:** 2025-12-15

**Authors:** Zhiqian Zhou, Yanjiao Lai, Xiping Lian, Yehui Tan, Wei Shi

**Affiliations:** 1 South China Sea Marine Biodiversity Collections / Guangdong Provincial Key Laboratory of Applied Marine Biology, South China Sea Institute of Oceanology, Chinese Academy of Sciences, Guangzhou, 510301, China South China Sea Institute of Oceanology, Chinese Academy of Sciences Guangzhou China; 2 University of Chinese Academy of Sciences, Beijing, 100049, China University of Chinese Academy of Sciences Beijing China; 3 South China Sea Development Research Institute, Ministry of Natural Resources (Remote Sensing Technology Application Center of South China Sea, NMR), Guangzhou 510300, China Ministry of Natural Resources Guangzhou China

**Keywords:** Copepods, *
Cymbasoma
*, South China Sea, taxonomy, zooplankton

## Abstract

A new monstrilloid copepod species, *Cymbasoma stricturum***sp. nov.**, is described and illustrated on the basis of adult females from the Pearl River estuary, northern South China Sea. The new species is closest to *C. morii* Sekiguchi, 1982, *C. sinopense* Üstün, Terbiyik & Suárez-Morales, 2014, and *C. jinigudira* Suárez-Morales & McKinnon, 2016, but it can be distinguished by a combination of characters including strongly protuberant, straight oral papilla; a cephalothorax that is distinctly constricted at the anterior two-fifths in both dorsal and lateral views; and two pairs of well-developed nipple-like processes on anterior dorsal surface, among other diagnostic traits. This is the ninth nominal species known in the *Cymbasoma longispinosum* species-group. We provide an updated dichotomous key for females and a revised worldwide distribution map of the species-group.

## ﻿Introduction

The order Monstrilloida Sars, 1901 is renowned for its peculiar life history strategies. These semiparasitic copepods undergo ontogenetic niche shift—from endoparasitic juveniles to planktonic free-living adults (Huys and Boxshall 1991). Adult specimens are characterized by non-feeding, free-swimming behaviour and the absence of mouthparts (Grygier and Ohtsuka 1995; Suárez-Morales 2011, 2018). Due to their unique life cycle, monstrilloids are rarely captured in routine zooplankton surveys, which results in significant gaps in our understanding of their biodiversity.

In recent decades, records of monstrilloids have increased substantially. A new genus, *Sarsimonstrillus* Suárez-Morales & McKinnon, 2025, was established by unique combination of characters, including paired uniramous horn-like processes between the antennule bases (Suárez-Morales and McKinnon 2025). Eight valid genera and approximately 197 accepted species are currently recognized within the single family Monstrillidae Dana, 1849 (Walter and Boxshall 2025). These species exhibit a wide distribution worldwide, but they are particularly numerous records in Australian waters (Suárez-Morales and McKinnon 2014, 2016, 2025). Currently, *Cymbasoma* Thompson, 1888 represents the most speciose genus, comprising about 44% of described species. New *Cymbasoma* species have been discovered recently in deep-sea habitats, where no previous records of Monstrilloida existed (Suárez-Morales and Mercado-Salas 2023).

Despite China’s possession of a 32,000-km coastline and vast maritime territories (Gao et al. 2013), taxonomic studies on monstrilloid copepods remain disproportionately scarce, with merely three novel species documented to date (Lian and Tan 2019; Lian and Zhou 2025; Zhou et al. 2025). The Pearl River estuary, located in southern China where the Pearl River discharges into the South China Sea, sustains rich marine biological communities and contributes significantly to regional fisheries. Our recent zooplankton survey in this estuary yielded a new species presenting long ovigerous spines. The taxonomic examination of this material revealed that it belongs to the *C. longispinosum* species-group.

This study aims to describe the new species through comparative analysis with its closest congeneric species, while providing a dichotomous key to the species-group based on foundational work by Suárez-Morales et al. (2020).

## ﻿Materials and methods

**Figure 1.**
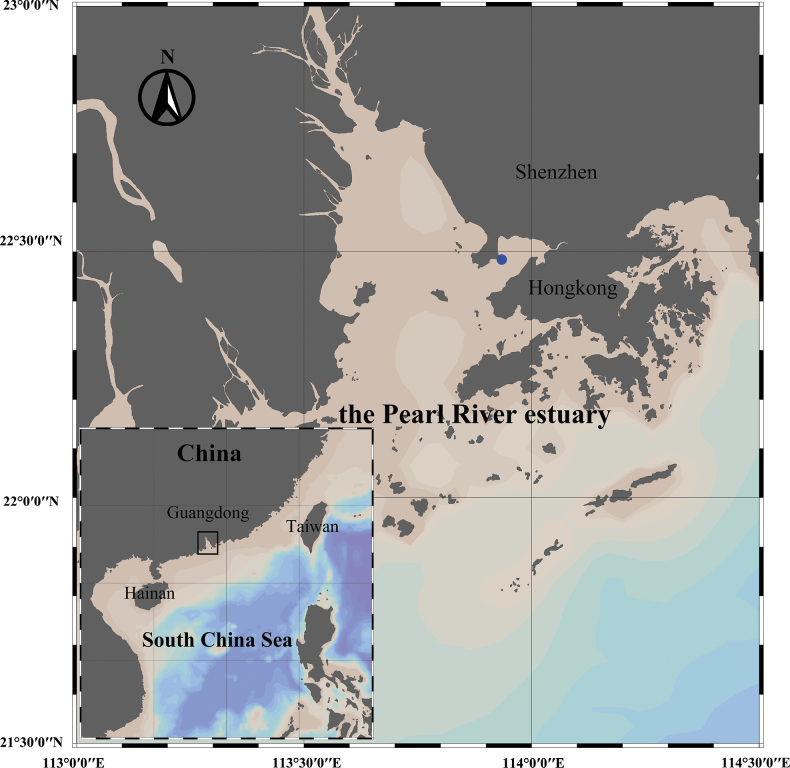
Sampling site (blue dot) of *Cymbasoma stricturum* sp. nov. near Shenzhen, Guangdong Province, China.

Zooplankton sample was collected from the coast near Shenzhen (22°29'3.5"N, 113°56'4.2"E), Guangdong Province, China on 7 February 2025 by a vertical tow net (505 μm mesh size, 0.8 m diameter at a towing speed of 0.5 m/s) from the surface to a depth of 5 m (Fig. 1). The specimens were immediately preserved in 5% formaldehyde. Observation and measurements were performed using a stereomicroscope (SMZ18, Nikon, Japan), and drawings were prepared based on the images captured with a digital camera (DS-Fi3, Nikon, Japan). Morphologic terminology follows Huys and Boxshall (1991). The nomenclature for the female monstrilloid antennulary armature proposed by Grygier and Ohtsuka (1995) is followed. The type specimen is deposited in the South China Sea marine biodiversity collections, Chinese Academy of Sciences (**SCSMBC**), Guangzhou.

## ﻿Taxonomy

### ﻿Subclass Copepoda Milne Edwards, 1840

**Order Monstrilloida Sars, 1901**

**Family Monstrillidae Dana, 1849**

**Genus *Cymbasoma* Thompson, 1888**

#### ﻿ Cymbasoma stricturum sp. nov.

Taxon classificationAnimaliaMonstrilloidaMonstrillidae

99783663-7C94-596E-A343-10DB74BBB280

https://zoobank.org/AD1FE586-06E3-4900-AEBC-9A59A9C28FB9

**Figure 2.**
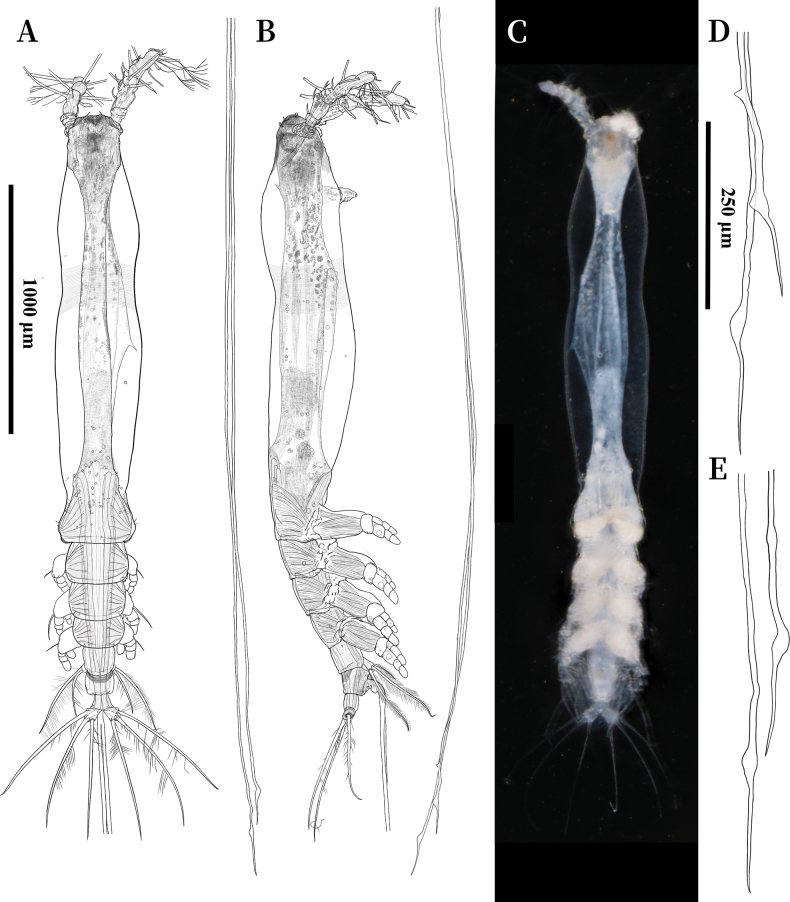
*Cymbasoma stricturum* sp. nov., female holotype. **A.** Habitus, dorsal; **B.** Habitus, lateral; **C.** Habitus, ventral; **D.** Distal of ovigerous spine, dorsal; **E.** Distal of ovigerous spine, lateral. **A–C.** Share the same scale bar; **D, E.** Share the same scale bar.

**Figure 3.**
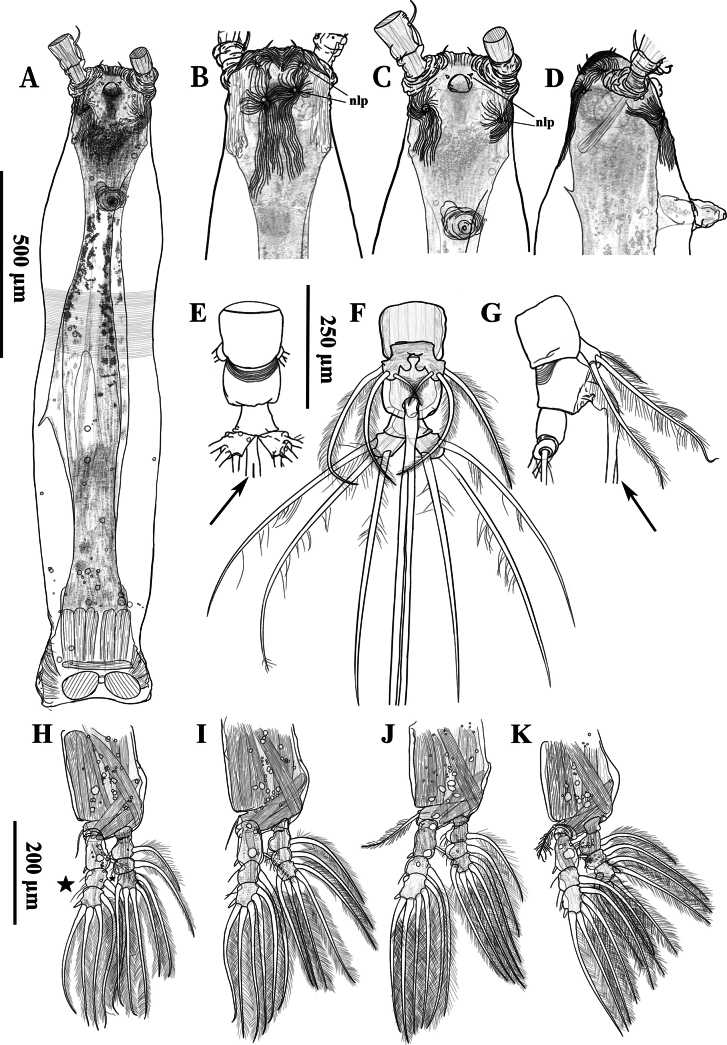
*Cymbasoma stricturum* sp. nov., female holotype. **A.** Cephalosome view, ventral; **B.** Anterior cephalothorax, emphasizing the striae of the surface, dorsal; **C.** Same, ventral; **D.** Same, lateral; **E.** Genital double and anal somites, lateral; **F.** Same, ventral; **G.** Same, lateral; **H–K.** Legs 1–4, nlp, nipple-like process. **B–G.** Share the same scale bar; **H–K.** Share the same scale bar.

**Figure 4.**
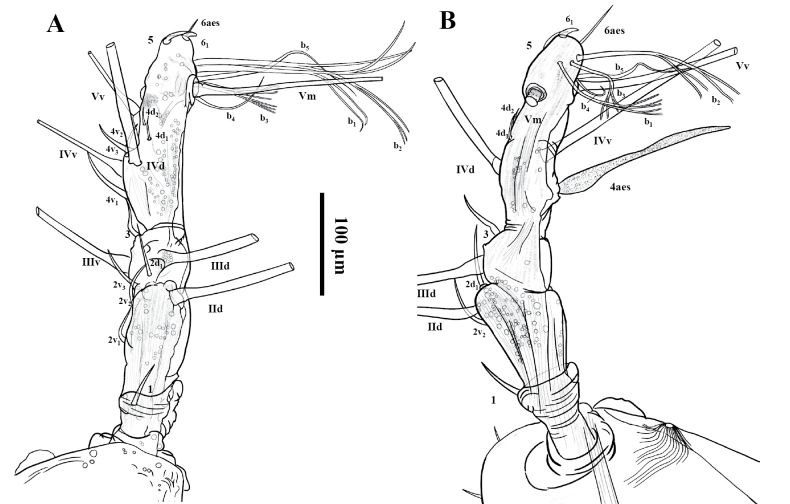
*Cymbasoma stricturum* sp. nov., female holotype. **A.** Left antennule, in dorsal view but tilted little from the medial side; **B.** Right antennule, lateral. **A, B.** Share the same scale bar.

Figs 2, 3, 4, Tables 1, 2

##### Type material.

***Holotype***: adult female (SCSMBC 240260); Yanjiao Lai leg.; 7 February 2025; partially dissected, formaldehyde preserved.

##### Type locality.

China • Guangdong Province; coast near Shenzhen; 22°29'3.5"N, 113°56'4.2"E; depth 5 m.

##### Etymology.

The new species name is derived from the Latin noun *strictūra*, meaning “constriction” or “narrowing,” in reference to the characteristic constricted region of the cephalothorax in the new species. The neuter ending –*um* is adopted (*stricturum*) to agree with the neuter gender of the generic name *Cymbasoma*. The proposed Chinese name is “缩缢舟形怪水蚤”.

##### Diagnosis.

Female *Cymbasoma* having the cephalothorax distinctly constricted laterally and ventrally at the anterior 2/5, bearing distinctive transverse belt-like striae at the same level; two pairs of well-developed nipple-like processes on anterior dorsal surface, both bearing shallow concentric reticulation (faintly visible but discernible upon specimen tilting). Antennule 4-segmented, short, and extending downwards, reaching 17.7% of total body length. Swimming legs 1–4 and leg 5 with well-developed plumose setae. Genital double-somite bearing transverse pattern of deep cuticular ridges on proximal dorsal surface. Caudal ramus rectangular, 1.3 times as long as wide, armed with three subequally long lightly setulated caudal setae. Ovigerous spines paired, long, 1.7 times as long as body length, spines proximally fused, with bifurcation beyond distal margin of caudal rami.

##### Description of adult female holotype.

Body elongate, with numerous oil droplets inside (Fig. 2A–C), 2.37 mm, measured from anterior end of cephalothorax to posterior margin of caudal rami, excluding antennules and caudal setae; cephalothorax (incorporating first pedigerous somite) mostly transparent, 1.70 mm long, representing 71% of total body length; oral papilla conical, straight, strongly protruding located ventrally at anterior 1/5 of cephalothorax (Fig. 2B); cephalothorax distinctly constricted at the anterior 2/5 in both dorsal and lateral views, bearing wide transverse belt of faint and shallow integumental striae (Figs 2A, B, 3A); pair of relatively large ocelli present, pigment cups moderately developed, medially conjoined, strongly pigmented; ventral cup indistinct; forehead flat, with conspicuous, medially convergent cuticular ridges between antennulary bases in dorsal view, bearing a pair of short, slender sensilla (Fig. 2A, B); esophagus within the cephalothorax, broad; ornamentation on anterior ventral surface: rounded cuticular protuberance and paired of simple, conical nipple-like processes between antennulary bases, without adjacent striae; a pair of well-developed nipple-like processes under antennulary bases, with conspicuous striae around (Fig. 3A, C); ornamentation on anterior dorsal surface: two pairs of well-developed nipple-like processes, both with faint and shallow concentric reticulation and three additional dorsal sensilla adjacent to these dorsal nipple-like processes (Fig. 3B–D).

Antennule short, extending downwards (Fig. 4A, B), representing almost 18% of total body length and 25% of cephalothorax length; antennule 4-segmented; relative length of segments, from proximal to distal as: 18.5; 21.5; 14.2; 45.8 = 100. In terms of the pattern described by Grygier and Ohtsuka (1995) for female monstrilloid antennulary armature, setae (Roman numerals) and spines (Arabic numerals), element 1 present on first segment; elements 2d_1_, 2v_1–3_, and setae IId present on second segment. Third segment with elements 3, setae IIId, and IIIv. Fourth segment long, representing 45.8% of antennule length; segment bearing elements 4d_1_, 4d_2_, 4v_1–3_; elements 4v_1_ well developed, thick, and remarkably long; setae IVd, IVv, Vv, Vm, and aesthetasc 4aes also present on same segment except Vd; element 5 spiniform, appressed in a subdistal position. Subterminal element 6_1_, 6aes present, element 6_2_ absent, element b_1–3_ branched and b_4,5_ unbranched (Fig. 4A, B).

Legs 1–4 (Fig. 3H–K) all with both endopod and exopod three-segmented; pedigerous somites 2–4 together accounting for 19.8% of total length in lateral view. Coxa without setae and lacking marginal rows of setae or spines. Basis not fully divided medially from coxa; basal seta of legs 3–4 with biserially plumose, that of leg 4 being thicker; seta on leg 3 being longest. Endopod segments 1–2 of legs 1–4 with swollen outer margins; exopod segment 3 of leg 1 and 3 bearing a small convex protuberance on outer margin. Ramus setae all biserially plumose except uniserially plumose outer seta on exopod segments 3; exopod segments 2–3 and endopod segments 1–2 with setules on outer margins (marked with an asterisk in Fig. 3H–K); outer distal spines on exopod segments 1 and 3 shorter than segments bearing them. Seta/spine armature of swimming legs 1–4 as in Table 1.

**Table 1.** Armature of legs 1–4 including coxa, basis, exopods, and endopods in *Cymbasoma stricturum* sp. nov. Roman numerals indicate numbers of spines, and Arabic numerals indicate numbers of setae.

	Coxa	Basis	Endopod	Exopod
Leg 1	0-0	1-0	0-1; 0-1; 2,2,1	I-1; 0-1;I,2,2
Leg 2–4	0-0	1-0	0-1; 0-1; 1,2,2	I-1; 0-1;I,2,3

Leg 5 bilobed, medially conjoined; inner (endopod) lobe rounded, not reaching the half-length of outer (exopod) lobe; outer lobe armed with two long setae apically and one subapical short seta, all heavily plumose; innermost seta relatively slender, shortest, not reaching the half-length of outer two (Fig. 3F, G).

Urosome consisting of three urosomites: fifth pedigerous somite, genital double-somite and anal somite, accounting for 10.5% of total body length, excluding caudal setae; length ratio of urosomites as: 43.3:34.4:22.3 (= 100); genital double-somite subquadrate, with transverse pattern of deep, transverse integumental ridges on proximal half of dorsal surface (Fig. 3E), ridges converging together in lateral surface (Fig. 3G); anal somite trapezoidal, smooth; Caudal rami subrectangular, 1.3 times as long as wide, armed with three subequally long lightly setulated caudal setae; ovigerous spines paired, 4.22 mm long, 1.7 times as long as body length (Fig. 2A–C); spines basally conjoined, individual spines arise beyond posterior margin of caudal rami (see the arrow in Fig. 3E–G); spines slender, straight at their base and along shaft, both with distally swollen sections and then tapering apically, one spine slightly shorter (Fig. 2D, E).

##### Remarks.

The monstrilloid copepod described herein from the Pearl River estuary is assigned to the genus *Cymbasoma* based on the presence of a single free somite between the caudal rami and the genital double-somite, and the caudal rami bearing only three setae (Huys and Boxshall 1991; Benz 2005; Suárez-Morales 2011). It is placed in the *Cymbasoma longispinosum* species-group by its body proportions—specifically the elongate cephalothorax (65–71% of total body length), long, proximally fused ovigerous spines (1.7 times the body length), and the presence of conspicuous cuticular dorsal ornamentation on the genital double-somite (Grygier 1994; Suárez-Morales 2011; Üstün et al. 2014; Suárez-Morales et al. 2020).

Since the original description of *C. longispinosum* s. str. from the English Channel (Bourne 1890), the nominal species has been reported worldwide (Giesbrecht 1893; Sars, 1921; Rose 1933; Dakin and Colefax 1940; Wilson 1950; Marques 1961; Martin-Thompson 1973; Dias 1996). However, subsequent studies revealed that some specimens initially identified as *C. longispinosum* represent undescribed cryptic species, including C. cf. longispinosum recorded from Brazil (Dias and Bonecker 2007; Leite et al. 2010; Suárez-Morales et al. 2020). These specimens exhibit subtle yet consistent differences (such as the proportions of the cephalothorax and genital double-somite), limited geographical distributions, and have consequently been classified under the *C. longispinosum* species-group (Suárez-Morales et al. 2020).

**Figure 5.**
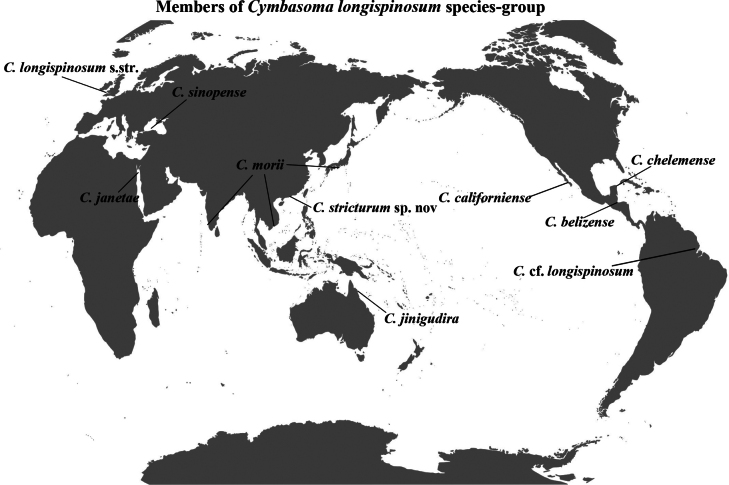
Worldwide distribution of valid species of the *Cymbasoma longispinosum* species-group. *C. longispinosum* s.str. conforms with Bourne’s (1890) description; the Brazilian C. cf. longispinosum is a tentative identification requiring further taxonomic verification (Dias and Bonecker 2007).

Eight species belonging to this group have been recognized worldwide (Fig. 5), including *C. longispinosum* (Bourne, 1890), *C. morii* Sekiguchi, 1982, *C. chelemense* Suárez-Morales & Escamilla, 1997, *C. californiense* Suárez-Morales & Palomares-García, 1999, *C. janetae* Mageed, 2010, *C. sinopense* Üstün, Terbiyik & Suárez-Morales, 2014, *C. jinigudira* Suárez-Morales & McKinnon, 2016, and *C. belizense* Suárez-Morales, Vásquez-Yeomans & Santoya, 2020 (Bourne 1890; Sekiguchi 1982; Suárez-Morales and Escamilla 1997; Suárez-Morales and Palomares-García 1998; Mageed 2010; Üstün et al. 2014; Suárez-Morales and McKinnon 2016; Suárez-Morales et al. 2020). The present species, *C. stricturum* sp. nov., constitutes the ninth one.

The new species most closely resembles *C. morii*, *C. sinopense*, and *C. jinigudira*, mainly by its relatively long cephalothorax (over 65% of body length) and ovigerous spines exceeding 150% of body length. *Cymbasoma stricturum* sp. nov. is readily distinguishable from its congeners by the following combination of characters:

1. Oral papilla strongly protuberant and straight, contrasting with the regularly developed condition in other members of the species group, except in *C. morii*, in which it is posteriorly curved.
2. Conspicuous, medially convergent forehead integumental ridges, which are absent in *C. morii*, longitudinally regular pattern in *C. sinopense*, shallow and simple transverse pattern in *C. jinigudira*.
3. Two pairs of well-developed nipple-like processes on anterior dorsal surface, both with shallow concentric reticulation that becomes discernible upon tilting the specimen; within the species group this character appears unique, although a similar condition has been described in the Mexican *C. quintanarroense* Suárez-Morales, 1994, which was originally described as belonging to the genus *Thaumaleus* (Suárez-Morales 1994).
4. Wide, transverse, belt-like striae, faint and shallow on anterior 2/5 of cephalothorax, shared only with *C. belizense* within the species group; distinguishingly, unlike *C. belizense*, the new species is uniquely characterized by a distinct transverse and ventral constriction of the cephalothorax at this same level; in addition, a similar but narrower belt-like striation is present in the Mexican *C. boxshalli* Suárez-Morales, 1993, originally described as *Thaumaleus boxshalli* (Suárez-Morales 1993).
5. Rounded ventral process between antennules, which is absent in *C. morii* and *C. jinigudira*, but shell-like in *C. sinopense*.
6. Armature of the outer lobe of leg 5, with all three setae being heavily plumose and the innermost seta not reaching the half-length of outer two. The strongly plumose condition is shared only with *C. longispinosum*; this contrasts with the sparsely plumose setae in *C. chelemense*, *C. californiense*, and *C. sinopense*, and with the naked setae in *C. morii*, *C. jinigudira*, and *C. belizense*.

Additionally, *C. stricturum* sp. nov. is among the largest members within the species-group based on key morphometric parameters: total body length (2.38 mm), cephalothorax–body length ratio (71%), and ovigerous spine length/body length ratio (1.7×). Notably, the plumose setae of legs 1–5 exhibit the most strongly developed observed among the known members of this species-group. Taken together, these characters confirm the recognition of *Cymbasoma stricturum* sp. nov. as a valid new species of *Cymbasoma*.

To facilitate interspecific comparisons, we critically re-evaluated the published diagnostic characters (Table 2) and synthesized a revised diagnostic key (Üstün et al. 2014; Suárez-Morales et al. 2020). Characters unverifiable from literature—notably the exopod/endopod length ratio of leg 5—were excluded. Illustrations of dissected fifth legs are rare, and the legs are usually depicted in situ from different angles.

**Table 2.** Comparative morphological and morphometric features of females of *Cymbasoma stricturum* sp. nov. and related species in the *Cymbasoma longispinosum* species-group. Sequence from A to E: *C. longispinosum* s.str., C. cf. longispinosum, *C. morii*, *C. chelemense*, *C. californiense*.

Character	Species									
	A	B	C	D	E	F	G	H	I	J
Pair of sensilla on forehead	—	—	yes	yes	no	—	no	no	no	yes
Cuticular ridges on forehead	—	no	no	yes	yes	no	yes	yes	yes	yes
Antennule/body length	27.7%	17.4%	16.3–17%	14.5%	17.5%	16%	14.6%	17%	22%	17%
Relative length of last antennular segment	—	50%	51%	51%	51%	51%	46%	49.6%	51.2%	45.8%
Ventral process present between antennules	—	yes	no	no	no	yes	yes	no	no	yes
Cephalothorax/total body length	—	64%	66–73%	68%	65%	71%	70%	69%	64%	71%
Wide transverse belt-like striae on cephalothorax	no	—	no	no	no	no	no	no	yes	yes
Oral papilla’s position along cephalothorax	15%	—	18–22%	19%	21%	17%	18%	17%	20%	20%
Cuticular ridges on genital double-somite	no	no	yes	yes	yes	yes	yes	yes	yes	yes
Posterior margin of genital double-somite straight in dorsal view	—	yes	no	yes	yes	no	yes	—	yes	yes
Genital double-somite with ventral anterior protuberance	yes	—	yes	yes	no	—	yes	no	no	yes
Genital double somite/anal somite length	—	2.2×	2×	1.7×	2×	1.8×	2×	—	—	1.6×
Ovigerous spines length/total length	1.43×	1.6×	1.5–2.4×	1.1–1.38×	1.3–1.43×	1.1×	1.8×	1.5×	—	1.7×
Point of bifurcation of ovigerous spines at distal end of caudal rami	no	no	yes	yes	yes	no	yes	yes	yes	yes
Protuberance on outer margin of leg 5 outer lobe	—	no	no	yes	yes	no	yes	yes	yes	no
Innermost seta of outer lobe on leg 5 shorter than others	yes	—	yes	yes	yes	yes	yes	yes	yes	yes
Innermost seta of outer lobe on leg 5 less than 50% of the others	no	—	yes	no	yes	*	no	yes	yes	yes
Total body length [mm]	2.3–3.16	1.8–2.4	1.9–3.2	2.3	2.1	1.78	2.5	2.82	2.33	2.37

– means no data. * means no data available from original description; it is mentioned that the inner seta is about half the length of the other two.

### ﻿Revised dichotomous key to females of the *Cymbasoma longispinosum* species-group

**Table:** 

1	Cuticular ridges present on forehead between antennule bases	**2**
–	Cuticular ridges absent, forehead surface between antennule bases smooth	**7**
2	Inner seta of leg 5 less than 50% of the others	**3**
–	Inner seta of leg 5 more than 50% of the others	**6**
3	Urosome cuticular striae on genital double somite only	**4**
–	Urosome cuticular striae on genital double, anal, and fifth pedigerous somites	***C. californiense***
4	Oral papilla strongly protuberant; anterior dorsal surface of cephalothorax with two pairs of nipple-like processes and three short sensilla	***C. stricturum* sp. nov.**
–	Oral papilla small or regularly developed condition; anterior dorsal surface of cephalothorax smooth	**5**
5	Innermost seta of outer lobe on leg 5 not reaching posterior margin of genital double-somite; inner lobe thumb-like; posterolateral corners of genital somite moderately produced, with field of deep ridges	***C. belizense***
–	Innermost seta of outer lobe on leg 5 reaching beyond genital somite margin; inner lobe small, globular; posterolateral corners of genital somite corners absent, smooth	***C. jinigudira***
6	Forehead ridges swirl-like pattern; medial ventral process between antennule bases absent, ovigerous spines relatively short, about as long as body	***C. chelemense***
–	Forehead ridges longitudinal; medial ventral process between antennule bases present, shell-like; ovigerous spines long, about 1.8 as long as body	***C. sinopense***
7	Posterior margin of genital double-somite straight in dorsal view	**8**
–	Posterior margin of genital double-somite clearly curved, convex in dorsal view	***C. morii***
8	Cephalothorax representing less than 66% of total body length; point of bifurcation of ovigerous spines not reaching distal end of caudal rami	**C. cf. longispinosum**
–	Cephalothorax representing more than 66% (usually 68–72%) of total body length; point of bifurcation of ovigerous spines beyond distal end of caudal rami	**9**
9	Point of bifurcation of ovigerous spines slightly beyond distal end of caudal rami; medial ventral process between antennule bases absent	***C. longispinosum* s.str.**
–	Point of bifurcation of ovigerous spines well beyond distal end of caudal rami; medial ventral process between antennule bases present, rounded	***C. janetae***

## Supplementary Material

XML Treatment for Cymbasoma stricturum

## ﻿Additional information

### Use of AI

No use of AI was reported.

### Author ORCIDs

Zhiqian Zhou https://orcid.org/0009-0006-8587-586X

Yanjiao Lai https://orcid.org/0000-0002-1936-6067

Xiping Lian https://orcid.org/0009-0002-3301-1999

Yehui Tan https://orcid.org/0000-0002-6378-2156

Wei Shi https://orcid.org/0009-0002-5486-2282

### Data availability

All of the data that support the findings of this study are available in the main text.
